# Performance of the Dexcom G7 Continuous Glucose Monitoring System in Pregnant Women with Diabetes

**DOI:** 10.1089/dia.2023.0516

**Published:** 2024-04-30

**Authors:** Sarit Polsky, Amy M. Valent, Elvira Isganaitis, Kristin Castorino, Grenye O'Malley, Stayce E. Beck, Peggy Gao, Lori M. Laffel, Florence M. Brown, Carol J. Levy

**Affiliations:** ^1^ Barbara Davis Center for Diabetes, University of Colorado Anschutz Medical Campus, Aurora, Colorado, USA.; ^2^ Oregon Health and Science University, Portland, Oregon, USA.; ^3^ Joslin Diabetes Center and Harvard Medical School, Boston, Massachusetts, USA.; ^4^ Sansum Diabetes Research Institute, Santa Barbara, California, USA.; ^5^ Icahn School of Medicine at Mount Sinai, New York, New York, USA.; ^6^ Department of Clinical Affairs, Dexcom, Inc., San Diego, California, USA.

**Keywords:** Continuous glucose monitoring, Accuracy, Safety, Pregnancy, Gestational Diabetes, Dexcom G7

## Abstract

**Background:**

We evaluated accuracy and safety of a seventh-generation real-time continuous glucose monitoring (CGM) system during pregnancy.

**Materials and Methods:**

Evaluable data for accuracy analysis were obtained from 96 G7 sensors (Dexcom, Inc.) worn by 96 of 105 enrolled pregnant women with type 1 (*n* = 59), type 2 (*n* = 21), or gestational diabetes (*n* = 25). CGM values were compared with arterialized venous glucose values from the YSI comparator instrument during 6-h clinic sessions at different time points throughout the sensors' 10-day wear period. The primary endpoint was the proportion of CGM values in the 70–180 mg/dL range within 15% of comparator glucose values. Secondary endpoints included the proportion of CGM values within 20% or 20 mg/dL of comparator values ≥ or <100 mg/dL, respectively (the %20/20 agreement rate).

**Results:**

Of the 1739 pairs with CGM in the 70–180 mg/dL range, 83.2% were within 15% of comparator values. The lower bound of the 95% confidence interval was 79.8%. Of the 2102 pairs with CGM values in the 40–400 mg/dL range, the %20/20 agreement rate was 92.5%. Of the 1659 pairs with comparator values in the 63–140 mg/dL range, the %20/20 agreement rate was 92.3%. The %20/20 agreement rates on days 1, 4 and 7, and 10 were 78.6%, 96.3%, and 97.3%, respectively. Consensus error grid analysis showed 99.8% of pairs in the clinically acceptable A and B zones. There were no serious adverse events. The sensors' 10-day survival rate was 90.3%.

**Conclusion:**

The G7 system is accurate and safe during pregnancies complicated by diabetes and does not require confirmatory fingerstick testing.

**Clinical Trial Registration:**

clinicaltrials.gov NCT04905628

## Introduction

At least 1 in 10 pregnancies in the United States is complicated by diabetes.^1,2^ Suboptimal glycemic control during pregnancy is associated with increased maternal morbidity and fetal morbidity and mortality.^3–5^ The risk for birth defects is largely mitigated by tight glycemic control^6,7^ as is the risk for adverse maternal, perinatal, and neonatal outcomes.^8–11^

The CONCEPTT study^12^ revealed the safety and clinical benefits of continuous glucose monitoring (CGM) in pregnant women with type 1 diabetes (T1D). The CONCEPTT study found that CGM was a cost-effective tool associated with significant improvements in the pregnancy-specific maternal time-in-target glucose range and clinically relevant reductions in the development of large-for-gestational age infants, severe neonatal hypoglycemia, and neonatal intensive care unit admissions >24 h.^12–14^

There are limited data on the use of CGM during pregnancies complicated by type 2 diabetes (T2D) or gestational diabetes mellitus (GDM) or on the benefit of newer more accurate CGM systems.^15^ Current CGM systems can display data locally as well as remotely, be used for insulin dosing decisions, automatically alert users (and their remote followers) to abnormal values or trends, and do not require routine calibration by the user. As such, CGM is increasingly used in the management of pregnancies complicated by diabetes.

Pregnancy is a dynamic period with constantly changing physiological, anatomic, and metabolic states. Accuracy of a sixth generation “G6” CGM system (Dexcom, Inc., San Diego, CA) was first described in nonpregnant people with diabetes by Shah et al.^16^ and Wadwa et al.^17^ In 2020, Castorino et al.^18^ provided G6 accuracy results in pregnant women with T1D, T2D, or GDM. The G6 system is not Food and Drug Administration (FDA) approved for use during pregnancy in the United States,^19^ but is approved for this indication in the European Union and the United Kingdom.

A seventh-generation “G7” CGM system (Dexcom) provides several improvements over the G6 system, including better accuracy in nonpregnant adults,^20^ a simpler insertion process,^21^ a shorter warmup period, and a smaller thinner wearable.^22^ The Dexcom G7 has received FDA clearance in the United States, where it is indicated for the management of diabetes in persons 2 years and older and can be worn during pregnancy for all types of diabetes. In Europe and the United Kingdom, G7 has secured CE mark and approval for people with diabetes of age 2 years and older, including pregnant women.^23^ In this study, we evaluated the accuracy and safety of the G7 system in pregnant women with T1D, T2D, or GDM.

## Methods

### Study design and participants

This prospective observational study was conducted at five investigational sites in the United States between July 21, 2021, and March 23, 2022. Inclusion criteria comprised age ≥18 years, singleton pregnancy, and a diagnosis of either T1D, T2D, or GDM. Exclusion criteria included hematocrit <30%, renal failure with dialysis, planned/scheduled magnetic resonance imaging or computed tomography studies, need for diathermy, or use of hydroxyurea. Participants were eligible for enrollment during all pregnancy trimesters.

The study was approved by central and local institutional review boards, and written informed consent was obtained before participation. This study was conducted in accordance with the provisions in applicable regulations and guidelines, including the Guidelines for Good Clinical Practice (GCP; ICH GCP [E6 (R2)]), the Declaration of Helsinki, and the general requirements outlined in ISO 14155:2011. Dexcom, Inc., provided funding, supplies, and technical expertise.

### Study protocol

Each participant was asked to wear two blinded G7 sensors, one on the back of each upper arm, for up to 10 days; the first sensor inserted was designated as the primary device. All 105 enrolled participants provided data for the safety analysis. In two participants, primary devices had out-of-box failures and were excluded from the accuracy analysis, leaving primary devices from 103 participants included in the survival analysis. Seven of the 103 participants in the full accuracy analysis sample either withdrew from the study (*n* = 2) or wore a primary sensor that failed (*n* = 5) before the scheduled in-clinic session, leaving a total of 96 participants whose primary devices contributed CGM data to the accuracy analysis described below.

### Outcome measurements

Accuracy of the primary devices was established during clinic sessions that involved sampling of venous blood (arterialized through heating pad applied distal to the antecubital IV catheter) at 15 ± 5 min intervals for 6 h for a maximum of 24 venous samples. Each participant participated in one clinic session, which occurred either near the beginning of the sensor wear period (day 1), the middle of sensor wear period (day 4 and 7), or the end of the sensor wear period (day 10) so that sufficient paired data points were collected to evaluate accuracy throughout the 10-day sensor life.

The clinic session time point was determined by participant preference and study need to obtain sufficient accuracy samples for sensor wear days 1, 4, 7, and 10. Moreover, blood sampling for the majority of the clinic sessions on day 1 was started within the first 4 h from the time of sensor insertion to minimize burden to the study participants. No intentional glucose manipulations were performed (such as hypoglycemia challenges or delayed insulin administration) given the clinical need to maintain safe glycemic control during pregnancy.

Participants continued their current diabetes management during the clinic session and were freely able to select meals and/or snacks for consumption during clinic sessions. Blood glucose concentrations were measured with the 2300 STAT PLUS glucose analyzer (YSI, Inc., Yellow Springs, OH). There was a total of 2102 matched CGM and venous glucose pairs available for analysis.

### Statistical analyses

The primary endpoint was defined as the percentage of CGM glucose values in the 70–180 mg/dL range that were within 15% of the corresponding YSI glucose values. The secondary endpoint was the %20/20 agreement rate, defined as the percentage of CGM values in the 40–400 mg/dL range that were within 20 mg/dL or 20% of corresponding YSI values that were <100 or ≥100 mg/dL, respectively. The other agreement rates were determined similarly to %20/20 but based on different percentages (i.e., %15/15, %30/30, %40/40) and the mean absolute relative difference (MARD), a measure used to assess the accuracy of CGM devices, were calculated. The one-sided 95% confidence interval (CI) lower bounds (LBs) and upper bounds were calculated using a 5000-iteration bootstrap method.

Additional analyses included plotting of the CGM–YSI paired values on a consensus (Parkes) error grid.^24^ The data availability rate was calculated as the number of valid CGM readings divided by the number of CGM readings expected; this was calculated for overall wear and for each individual day a sensor was worn. The survival rate was calculated using the number of sensor failures over the 10-day wear period. Safety was assessed as the number of reported device-related adverse events.

## Results

Demographic and baseline characteristics of the 105 enrolled participants appear in Table 1. There were 1739 CGM–YSI matched pairs with CGM readings in the 70–180 mg/dL range that were used in the primary endpoint analysis and 2102 CGM readings in the 40–400 mg/dL range that were used in the secondary endpoint analysis. For the primary endpoint, 83.2% of the CGM values were within 15% of the comparator (YSI) value, and the LB of this proportion's one-sided 95% CI was 79.8%. For the secondary endpoint, 92.5% of the CGM values met the %20/20 criteria, and the LB of the 95% CI was 89.7%.

**Table 1.** Participant Demographics and Baseline Characteristics (*n* = 105) at Entry into the Study

Attribute	Value
Age, years	
Mean (SD)	32.6 (5.2)
Race, *n* (%)	
White	78 (74.3)
Asian	8 (7.6)
Native Hawaiian or Pacific Islander	1 (1.0)
Black, African American, or African Heritage	7 (6.7)
Other	5 (4.8)
Decline to answer	6 (5.7)
Ethnicity, *n* (%)^a^	
Hispanic or Latino	27 (26.2)
BMI category (kg/m^2^), *n* (%)^b^	
<18.5	0 (0.0)
18.5–<25	18 (17.3)
25–<30	32 (30.8)
30–35	30 (28.8)
>35	24 (23.1)
Parity, *n*	
Median (IQR)	1.0 (0.0–1.0)
Trimester, *n* (%)	
1st	17 (16.2)
2nd	54 (51.4)
3rd	34 (32.4)
Type of diabetes, *n* (%)	
Type 1	59 (56.2)
Type 2	21 (20.0)
Gestational	25 (23.8)
Diabetes duration, years^c^	
Mean (SD)	12.8 (9.3)
Median (range)	13.5 (0.0–33.0)
A1C, %	
Mean (SD)	6.0 (1.1)
Median (range)	5.7 (4.5–10.6)

^a^ Two participants had missing ethnicity data.

^b^ One participant had missing BMI data.

^c^ Excludes participants with GDM.

BMI, body mass index; GDM, gestational diabetes mellitus; IQR, interquartile range; SD, standard deviation.

The overall MARD was 9.5% (Table 2) and was noted to be higher on day 1. The overall mean bias was −0.9 mg/dL (Table 3). Analysis of 1659 CGM–YSI matched pairs in the comparator glucose range of 63–140 mg/dL, the target range for pregnant women with T1D, showed 84.2% of the CGM values within %15/15 of the comparator value and 92.3% within %20/20 (Table 3). Considering the 1395 matched pairs with comparator values in the narrower range of 63–120 mg/dL range, the %20/20 agreement rate was 93.1%.

**Table 2.** Primary, Secondary, and Other Endpoints

Attribute	Value
15% (95% LB), %^a^	83.2 (79.8)
%15/15 (95% LB), %	85.0 (81.3)
%20/20 (95% LB), %^b^	92.5 (89.7)
%30/30 (95% LB), %	97.6 (96.0)
%40/40 (95% LB), %	98.9 (97.8)
MARD (95% UB), %	9.5 (10.5)

^a^ Primary endpoint; analysis restricted to 1739 matched pairs with CGM values in the 70–180 mg/dL range.

^b^ Secondary endpoint. Among secondary and other endpoints, agreement rates were calculated with 2102 matched pairs with CGM values in the 40–400 mg/dL range using absolute relative differences for YSI values ≥100 mg/dL and absolute differences for YSI values <100 mg/dL.

LB, lower bound; MARD, mean absolute relative difference; UB, upper bound.

**Table 3.** Accuracy by Comparator Glucose Range

Comparator glucose range (mg/dL)	Matched pairs (*n*)	%15/15 (%)	%20/20 (%)	%30/30 (%)	%40/40 (%)	Mean bias (mg/dL)
<70	140	84.3	92.1	97.1	98.6	4.9
63–140	1659	84.2	92.3	97.7	99.0	−0.7
70–180	1799	84.2	92.2	97.5	98.8	−1.3
>180	163	94.5	96.9	99.4	100	−1.1
Overall	2102	85.0	92.5	97.6	98.9	−0.9

**FIG. 1.**
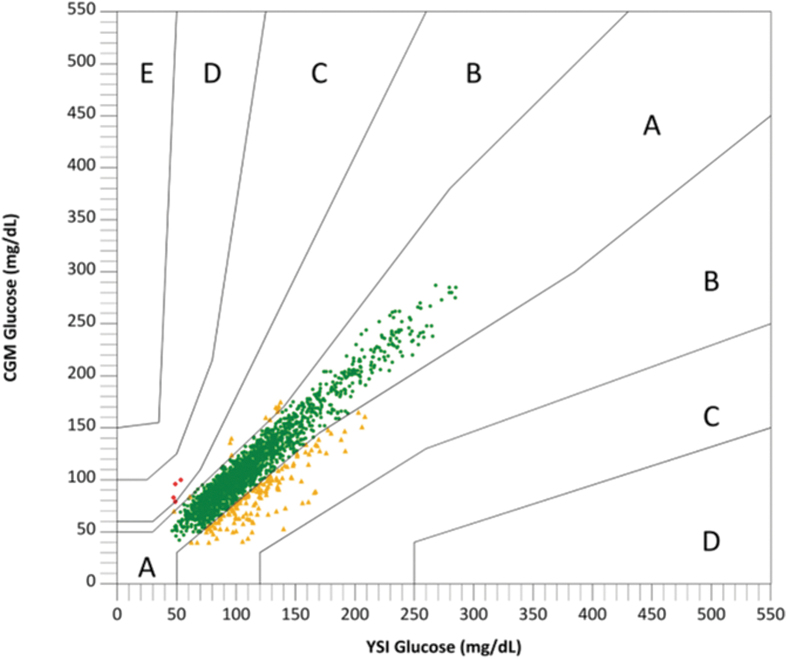
Secondary endpoint: consensus (Parkes) error grid. Consensus error grid analysis of glucose values from the G7 CGM system versus YSI plasma glucose values (pooled data for 96 participants with 2102 YSI–CGM pairs). Green, yellow, and red dots are in Zones A, B, and C, respectively.

Parkes error grid analysis (Fig. 1) of 2102 matched pairs showed that 2098 (99.8%) matched pairs were in either Zone A (clinically accurate, no effect on clinical action) or Zone B (altered clinical action with little or no effect on clinical outcome). The overall data availability rate for days 1–10 was 99.6% and ranged from 99.1% (observed on days 1 and 10) to 99.9% (observed on days 2 and 3). The 10-day survival rate was 90.3%. Of the nine sensors that failed early, four had adhesive failures, two were pulled out early, and three experienced early sensor shutoffs. Consistent with other populations and devices, the accuracy was lowest on day 1 and highest on day 10. The %20/20 values were 78.6% on day 1 of sensor wear, 96.3% on days 4 and 7, and 97.3% on day 10.

Exploratory analysis was conducted on subsets of data stratified by diabetes type, gestational age, HbA1C, and body mass index (BMI). Although not designed or powered to rule out between-group differences with respect to these variables, analysis of %20/20 agreement rates revealed no statistically significant accuracy differences between participants with T1D, T2D, or GDM, and no statistically significant accuracy differences at different gestational ages, different HbA1c levels, or different BMI values (data not shown).

The overall percentage of CGM readings in the pregnancy-specific time-in-range of 63–140 mg/dL was 71.8% and varied with respect to diabetes type (T1D: 64.8%; T2D: 68.9%; GDM, 91.1%). With respect to device safety, seven nonserious adverse events were reported, two of which were related to the device or study: one episode of bleeding at the sensor insertion site and one vasovagal event associated with sensor insertion. No skin irritation, local infections, excessive pain or discomfort, or serious adverse events were reported.

## Conclusions

This study demonstrated that the Dexcom G7 CGM system provided accurate readings in pregnant individuals with diabetes. The overall %15/15 and %20/20 agreement rates were 85.0% and 92.5%, respectively, providing confidence in clinical use of the G7 system during pregnancy. These results are similar to G7 accuracy in nonpregnant adults (%15/15 and %20/20 agreement rates of 89.6% and 95.3%, respectively)^20^ and children as young as 2 years old along with adolescents (%15/15 and %20/20 agreement rates of 88.8% and 95.3%, respectively)^25^ with diabetes.

As reported previously,^18^ arm-placed G6 sensors used during pregnancy had a %15/15 agreement rate of 86.1% and a %20/20 agreement rate of 95.9%; these agreement rates are comparable with those of G7 sensors reported here. The Dexcom G7 sensors were well tolerated during all trimesters of pregnancy, and there were no serious adverse events.

A limitation of our study is its lack of intentional glucose manipulations given the health needs of the sample of pregnant women with diabetes participating, resulting in only 6.7% of the matched pairs in hypoglycemia <70 mg/dL, even fewer in the <63 mg/dL range, and only 7.8% in hyperglycemia >180 mg/dL. The large majority (86%) of the matched pairs were in the 70–180 mg/dL target range, and most (75%) were in the tighter 70–140 mg/dL range. Preservation of near-euglycemia was important to avoid jeopardizing participant and fetal safety.^26^

A second limitation is that all data were from sensors worn on the back of the upper arm, preventing analysis of performance at other wear sites. In the United States, the back of the upper arm is the only approved wear location for people older than 6 years. Additional limitations are that the study was not designed or powered to rule out accuracy differences based on diabetes type, gestational age, HbA1c, BMI, or pregnancy conditions such as labor, gestational hypertensive disorders, infections, or lactation that further alter the physiological state. Also, blood sampling for most of the clinic sessions on day 1 was started within the first 4 h from the time of sensor insertion, which likely impacted day 1 accuracy.

It also should be noted that there were fewer participants in the first and third trimesters. Strengths of the study include its use of the YSI instrument for comparator values and separate analysis of data from different glucose concentration ranges. Data were obtained from 96 pregnant persons and 2102 matched pairs in this study, which contrasts with the 32 women and 734 matched pairs analyzed in an earlier study^18^ of the G6 system during pregnancy.

In conclusion, the Dexcom G7 CGM System is safe and accurate without confirmatory fingerstick testing in pregnant individuals with T1D, T2D, and GDM. Despite advances in diabetes technologies, relatively few devices are approved for use in pregnant individuals, and studies demonstrating the accuracy of these devices across gestational ages are lacking; thus, this study fulfills a significant unmet medical need. CGM data during pregnancy contribute to improvements in maternal and neonatal outcomes in pregnancies complicated by diabetes. Additional studies are needed to evaluate long-term device use, maternal experiences, and maternal and neonatal outcomes in real-world settings.
